# Dichloridobis[2-methyl­sulfanyl-4-(pyridin-2-yl)pyrimidine-κ^2^ *N* ^3^,*N* ^4^]cobalt(II)

**DOI:** 10.1107/S1600536811030881

**Published:** 2011-08-06

**Authors:** Wen-Na Yang

**Affiliations:** a School of Chemistry and Chemical Engineering, Southeast University, Nanjing 211189, People’s Republic of China

## Abstract

The asymmetric unit of the title compound, [CoCl_2_(C_10_H_9_N_3_S)_2_], contains one half-mol­ecule with the Co^II^ atom situtated on a twofold rotational axis. The Co^II^ atom, in an octa­hedral enviroment, is coordinated by four N atoms from two 2-methyl­sulfanyl-4-(pyridin-2-yl)pyrimidine ligands and two Cl atoms.

## Related literature

For coordination compounds derived from the prototypical ligands 4-(pyridin-*n*-yl)pyrimidine-2-thiol (*n* = 2, 3, 4) and their S-modified derivatives reported by our group, see: Huang *et al.* (2007 ▶); Dong *et al.* (2009 ▶); Zhu *et al.* (2009 ▶). 
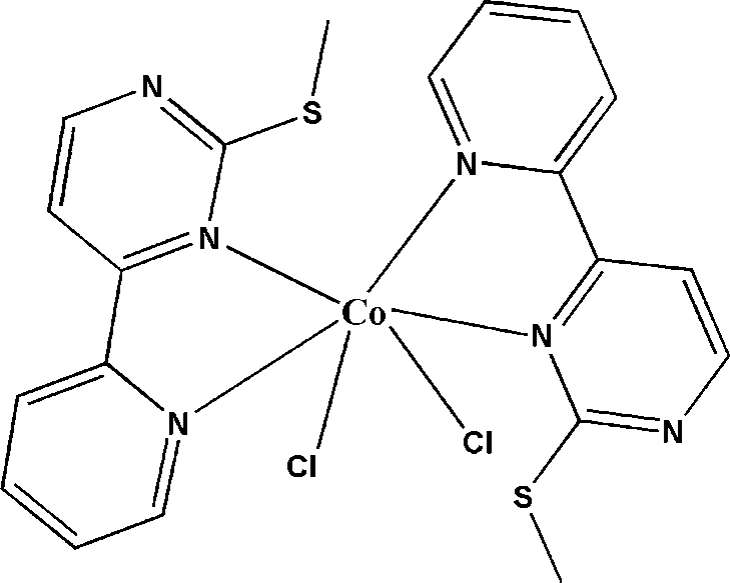


## Experimental

### 

#### Crystal data

[CoCl_2_(C_10_H_9_N_3_S)_2_]
*M* *_r_* = 536.37
Monoclinic, 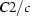

*a* = 8.709 (11) Å
*b* = 17.10 (2) Å
*c* = 15.328 (19) Å
β = 102.34 (3)°
*V* = 2230 (5) Å^3^
*Z* = 4
Mo *K*α radiation
μ = 1.22 mm^−1^
*T* = 298 K
0.35 × 0.28 × 0.24 mm

#### Data collection

Bruker APEXII CCD area-detector diffractometer
Absorption correction: multi-scan (*SADABS*; Bruker, 2001 ▶) *T*_min_ = 0.671, *T*_max_ = 0.747
6138 measured reflections
2586 independent reflections
1636 reflections with *I* > 2σ(*I*)
*R*_int_ = 0.052

#### Refinement

*R*[*F* ^2^ > 2σ(*F* ^2^)] = 0.045
*wR*(*F* ^2^) = 0.060
*S* = 0.93
2586 reflections
141 parameters
H-atom parameters constrained
Δρ_max_ = 0.33 e Å^−3^
Δρ_min_ = −0.50 e Å^−3^

### 

Data collection: *APEX2* (Bruker, 2007 ▶); cell refinement: *SAINT-Plus* (Bruker, 2007 ▶); data reduction: *SAINT-Plus*; program(s) used to solve structure: *SHELXS97* (Sheldrick, 2008 ▶); program(s) used to refine structure: *SHELXL97* (Sheldrick, 2008 ▶); molecular graphics: *SHELXTL* (Sheldrick, 2008 ▶); software used to prepare material for publication: *SHELXTL*.

## Supplementary Material

Crystal structure: contains datablock(s) I, global. DOI: 10.1107/S1600536811030881/vm2113sup1.cif

Structure factors: contains datablock(s) I. DOI: 10.1107/S1600536811030881/vm2113Isup2.hkl

Additional supplementary materials:  crystallographic information; 3D view; checkCIF report

## Figures and Tables

**Fig. 1.**
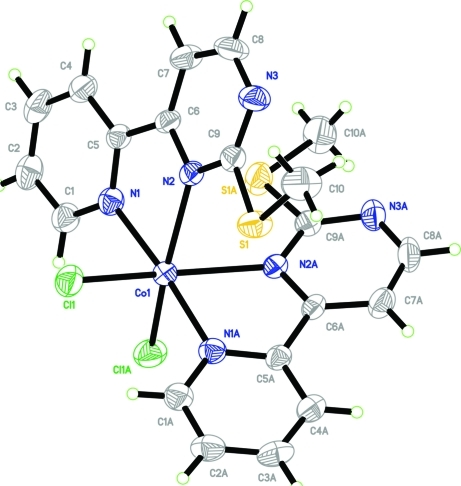
The structure of the title compound with displacement ellipsoids drawn at the 50% probability level; Atoms labeled with flag A are generated by symmetry operation -x + 1, y, -z + 1/2.

## supplementary crystallographic information

### Comment

Over the past few years, a number of coordination compounds derived from the prototypical ligands of 4-(pyridin-n-yl)pyrimidine-2-thiol (n = 2, 3, 4) and their S-modified derivatives have been reported by our group (Huang *et al.*, 2007; Dong *et al.*, 2009; Zhu *et al.*, 2009). In light of our previous study, it is apparent that the coordination chemistry of such type of ligands is profoundly effected by the nature of the S atom. As part of our research program, we present here a discrete Co^II^ coordination compound with ligand *L* (*L* = 2-(methylthio)-4-(pyridin-2-yl)pyrimidine) bearing a methylthio group.

The asymmetric unit of the title compound owns half of one molecule. A twofold rotational axis passes through the Co^II^ atom. As depicted in Fig. 1, the Co^II^ atom in the title compound is surrounded by two *L* ligands and two Cl atoms, adopting an octahedral coordination geometry. Like 2,2'-bipyridine, the ligand *L* serves as a chelating ligand through two N atoms with Co—N bond distances being 2.148 (3)Å and 2.299 (3) Å, whilst two Cl atoms are bound to the Co^II^ atom in *cis* positions with equal Co—Cl bond distance (2.434 Å). In *L*, the pyridyl and pyrimidinyl rings are twisted by a dihedral angle of 9.9 (1)°, and the methylthio group is not involved into the metal coordination as a result of the weak affinity of the S atom for Co.

### Experimental

The mixture of CoCl_2_ (0.1 mmol) and *L* (0.2 mmol) in 10 ml of ethanol was stirred for 20 min at room temperature. After filtration, the mother solution was allowed to stand for one week to give blue crystals suitable for X-ray diffraction analysis.

### Refinement

All H atoms bounded to C atoms were positioned geometrically and allowed to ride on their parent atoms, with C—H = 0.93Å (CH) and *U*_iso_(H) = 1.2*U*_eq_(C), C—H = 0.97Å (CH_3_) and *U*_iso_(H) = 1.5*U*_eq_(C).

### Crystal data

**Table:**

[CoCl_2_(C_10_H_9_N_3_S)_2_]	*F*(000) = 1092
*M**_r_* = 536.37	*D*_x_ = 1.598 Mg m^−^^3^
Monoclinic, *C*2/*c*	Mo *K*α radiation, λ = 0.71073 Å
Hall symbol: -C 2yc	Cell parameters from 2588 reflections
*a* = 8.709 (11) Å	θ = 2.3–25.5°
*b* = 17.10 (2) Å	µ = 1.22 mm^−^^1^
*c* = 15.328 (19) Å	*T* = 298 K
β = 102.34 (3)°	Block, blue
*V* = 2230 (5) Å^3^	0.35 × 0.28 × 0.24 mm
*Z* = 4	

### Data collection

**Table:**

Bruker APEXII CCD area-detector diffractometer	2586 independent reflections
Radiation source: fine-focus sealed tube	1636 reflections with *I* > 2σ(*I*)
graphite	*R*_int_ = 0.052
φ and ω scans	θ_max_ = 28.4°, θ_min_ = 2.7°
Absorption correction: multi-scan (*SADABS*; Bruker, 2001)	*h* = −11→9
*T*_min_ = 0.671, *T*_max_ = 0.747	*k* = −21→17
6138 measured reflections	*l* = −14→20

### Refinement

**Table:**

Refinement on *F*^2^	Primary atom site location: structure-invariant direct methods
Least-squares matrix: full	Secondary atom site location: difference Fourier map
*R*[*F*^2^ > 2σ(*F*^2^)] = 0.045	Hydrogen site location: inferred from neighbouring sites
*wR*(*F*^2^) = 0.060	H-atom parameters constrained
*S* = 0.93	*w* = 1/[σ^2^(*F*_o_^2^) + (0.0104*P*)^2^] where *P* = (*F*_o_^2^ + 2*F*_c_^2^)/3
2586 reflections	(Δ/σ)_max_ < 0.001
141 parameters	Δρ_max_ = 0.33 e Å^−^^3^
0 restraints	Δρ_min_ = −0.50 e Å^−^^3^

### Special details

**Table:**

Geometry. All e.s.d.'s (except the e.s.d. in the dihedral angle between two l.s. planes) are estimated using the full covariance matrix. The cell e.s.d.'s are taken into account individually in the estimation of e.s.d.'s in distances, angles and torsion angles; correlations between e.s.d.'s in cell parameters are only used when they are defined by crystal symmetry. An approximate (isotropic) treatment of cell e.s.d.'s is used for estimating e.s.d.'s involving l.s. planes.
Refinement. Refinement of *F*^2^ against ALL reflections. The weighted *R*-factor *wR* and goodness of fit *S* are based on *F*^2^, conventional *R*-factors *R* are based on *F*, with *F* set to zero for negative *F*^2^. The threshold expression of *F*^2^ > σ(*F*^2^) is used only for calculating *R*-factors(gt) *etc*. and is not relevant to the choice of reflections for refinement. *R*-factors based on *F*^2^ are statistically about twice as large as those based on *F*, and *R*- factors based on ALL data will be even larger.

### Fractional atomic coordinates and isotropic or equivalent isotropic displacement parameters (Å^2^)

**Table:**

	*x*	*y*	*z*	*U*_iso_*/*U*_eq_	
Co1	0.5000	0.29835 (3)	0.2500	0.02888 (15)	
Cl1	0.56198 (8)	0.38235 (4)	0.13403 (4)	0.0435 (2)	
S1	0.76922 (8)	0.15867 (5)	0.18274 (4)	0.0419 (2)	
N2	0.4663 (2)	0.19418 (12)	0.15296 (11)	0.0274 (5)	
N1	0.2595 (2)	0.30314 (13)	0.17840 (12)	0.0309 (5)	
C6	0.3142 (3)	0.18317 (15)	0.10981 (14)	0.0279 (6)	
C9	0.5705 (3)	0.13805 (16)	0.13928 (15)	0.0314 (7)	
C5	0.2020 (3)	0.24704 (16)	0.11833 (14)	0.0288 (6)	
C7	0.2671 (3)	0.11737 (17)	0.05868 (16)	0.0389 (7)	
H7A	0.1634	0.1105	0.0285	0.047*	
C1	0.1648 (3)	0.36330 (17)	0.18607 (16)	0.0381 (7)	
H1A	0.2041	0.4035	0.2255	0.046*	
N3	0.5331 (3)	0.07212 (14)	0.09342 (13)	0.0402 (6)	
C2	0.0109 (3)	0.36861 (17)	0.13814 (17)	0.0422 (8)	
H2A	−0.0516	0.4109	0.1463	0.051*	
C8	0.3816 (3)	0.06227 (17)	0.05450 (17)	0.0440 (8)	
H8A	0.3519	0.0162	0.0232	0.053*	
C4	0.0501 (3)	0.24955 (17)	0.06704 (15)	0.0364 (7)	
H4A	0.0145	0.2105	0.0254	0.044*	
C10	0.8661 (3)	0.07695 (18)	0.14226 (19)	0.0572 (9)	
H10A	0.9779	0.0822	0.1622	0.086*	
H10B	0.8396	0.0762	0.0782	0.086*	
H10C	0.8323	0.0291	0.1649	0.086*	
C3	−0.0481 (3)	0.31061 (18)	0.07843 (17)	0.0424 (8)	
H3B	−0.1516	0.3123	0.0464	0.051*	

### Atomic displacement parameters (Å^2^)

**Table:**

	*U*^11^	*U*^22^	*U*^33^	*U*^12^	*U*^13^	*U*^23^
Co1	0.0301 (3)	0.0267 (3)	0.0278 (2)	0.000	0.0016 (2)	0.000
Cl1	0.0447 (5)	0.0451 (5)	0.0379 (4)	−0.0040 (4)	0.0027 (3)	0.0098 (3)
S1	0.0323 (4)	0.0441 (6)	0.0470 (4)	0.0022 (4)	0.0030 (4)	−0.0135 (3)
N2	0.0295 (13)	0.0292 (14)	0.0223 (9)	0.0010 (11)	0.0029 (10)	0.0001 (9)
N1	0.0313 (13)	0.0318 (15)	0.0296 (10)	0.0039 (12)	0.0069 (10)	0.0007 (10)
C6	0.0299 (16)	0.0316 (18)	0.0211 (11)	−0.0022 (13)	0.0029 (12)	−0.0015 (11)
C9	0.0344 (16)	0.0310 (19)	0.0278 (12)	0.0009 (14)	0.0046 (12)	0.0004 (11)
C5	0.0316 (16)	0.0309 (17)	0.0233 (12)	−0.0039 (14)	0.0044 (12)	0.0029 (11)
C7	0.0306 (16)	0.045 (2)	0.0376 (15)	−0.0031 (16)	−0.0006 (13)	−0.0075 (13)
C1	0.0410 (18)	0.037 (2)	0.0349 (14)	0.0062 (16)	0.0057 (14)	−0.0009 (12)
N3	0.0412 (16)	0.0333 (17)	0.0426 (13)	0.0021 (13)	0.0015 (12)	−0.0111 (11)
C2	0.0423 (19)	0.047 (2)	0.0384 (15)	0.0148 (17)	0.0102 (15)	0.0071 (14)
C8	0.047 (2)	0.033 (2)	0.0492 (17)	−0.0070 (17)	0.0031 (16)	−0.0157 (14)
C4	0.0339 (17)	0.041 (2)	0.0322 (14)	−0.0013 (15)	0.0022 (13)	0.0014 (13)
C10	0.0378 (19)	0.059 (2)	0.072 (2)	0.0117 (18)	0.0058 (16)	−0.0197 (17)
C3	0.0294 (16)	0.055 (2)	0.0398 (15)	0.0046 (16)	0.0017 (14)	0.0122 (15)

### Geometric parameters (Å, °)

**Table:**

Co1—N1^i^	2.148 (3)	C5—C4	1.388 (4)
Co1—N1	2.148 (3)	C7—C8	1.383 (4)
Co1—N2	2.299 (3)	C7—H7A	0.9300
Co1—N2^i^	2.299 (3)	C1—C2	1.387 (4)
Co1—Cl1^i^	2.434 (2)	C1—H1A	0.9300
Co1—Cl1	2.434 (2)	N3—C8	1.337 (3)
S1—C9	1.752 (3)	C2—C3	1.373 (4)
S1—C10	1.810 (3)	C2—H2A	0.9300
N2—C6	1.361 (3)	C8—H8A	0.9300
N2—C9	1.368 (3)	C4—C3	1.384 (4)
N1—C5	1.350 (3)	C4—H4A	0.9300
N1—C1	1.340 (3)	C10—H10A	0.9600
C6—C7	1.382 (4)	C10—H10B	0.9600
C6—C5	1.490 (4)	C10—H10C	0.9600
C9—N3	1.332 (3)	C3—H3B	0.9300
			
N1^i^—Co1—N1	175.62 (12)	N1—C5—C4	122.3 (2)
N1^i^—Co1—N2	109.62 (8)	N1—C5—C6	115.2 (2)
N1—Co1—N2	73.94 (8)	C4—C5—C6	122.5 (2)
N1^i^—Co1—N2^i^	73.94 (8)	C8—C7—C6	116.8 (3)
N1—Co1—N2^i^	109.62 (8)	C8—C7—H7A	121.6
N2—Co1—N2^i^	78.41 (14)	C6—C7—H7A	121.6
N1^i^—Co1—Cl1^i^	87.05 (8)	N1—C1—C2	123.2 (3)
N1—Co1—Cl1^i^	90.36 (8)	N1—C1—H1A	118.4
N2—Co1—Cl1^i^	155.81 (5)	C2—C1—H1A	118.4
N2^i^—Co1—Cl1^i^	90.14 (11)	C9—N3—C8	116.7 (2)
N1^i^—Co1—Cl1	90.36 (8)	C3—C2—C1	119.2 (3)
N1—Co1—Cl1	87.05 (8)	C3—C2—H2A	120.4
N2—Co1—Cl1	90.14 (11)	C1—C2—H2A	120.4
N2^i^—Co1—Cl1	155.81 (5)	N3—C8—C7	123.1 (3)
Cl1^i^—Co1—Cl1	107.66 (10)	N3—C8—H8A	118.5
C9—S1—C10	102.08 (15)	C7—C8—H8A	118.5
C6—N2—C9	115.9 (2)	C5—C4—C3	119.4 (3)
C6—N2—Co1	113.46 (15)	C5—C4—H4A	120.3
C9—N2—Co1	130.23 (17)	C3—C4—H4A	120.3
C5—N1—C1	117.4 (2)	S1—C10—H10A	109.5
C5—N1—Co1	120.02 (17)	S1—C10—H10B	109.5
C1—N1—Co1	122.42 (19)	H10A—C10—H10B	109.5
N2—C6—C7	121.9 (2)	S1—C10—H10C	109.5
N2—C6—C5	116.4 (2)	H10A—C10—H10C	109.5
C7—C6—C5	121.7 (3)	H10B—C10—H10C	109.5
N3—C9—N2	125.4 (2)	C4—C3—C2	118.4 (3)
N3—C9—S1	118.8 (2)	C4—C3—H3B	120.8
N2—C9—S1	115.7 (2)	C2—C3—H3B	120.8

Symmetry codes: (i) −*x*+1, *y*, −*z*+1/2.
